# Development and validation of a model for individualized prediction of cervical insufficiency risks in patients undergoing IVF/ICSI treatment

**DOI:** 10.1186/s12958-020-00693-x

**Published:** 2021-01-07

**Authors:** Yaoqiu Wu, Xiaoyan Liang, Meihong Cai, Linzhi Gao, Jie Lan, Xing Yang

**Affiliations:** 1 Reproductive Medicine Center, The Sixth Affiliated Hospital, Sun Yat-sen University, Guangzhou, 510655 Guangdong China; 2 Reproductive Medicine Center, The Sun Yat-sen Memorial Hospital, Sun Yat-sen University, Guangzhou, 510120 Guangdong China; 3 Reproductive Medicine Centre, Guangzhou First People’s Hospital, School of Medicine, South China University of Technology, Guangzhou, 51000 Guangdong China

**Keywords:** Cervical insufficiency, Androgen excess, Nomogram, Prediction models, Pregnancy

## Abstract

**Background:**

Women who conceived with in vitro fertilization (IVF) or intracytoplasmic sperm injection (ICSI) are more likely to experience adverse pregnancy outcomes than women who conceived naturally. Cervical insufficiency (CI) is one of the important causes of miscarriage and premature birth, however there is no published data available focusing on the potential risk factors predicting CI occurrence in women who received IVF/ICSI treatment. This study aimed to identify the risk factors that could be integrated into a predictive model for CI, which could provide further personalized and clinically specific information related to the incidence of CI after IVF/ICSI treatment.

**Patients and methods:**

This retrospective study included 4710 patients who conceived after IVF/ICSI treatment from Jan 2011 to Dec 2018 at a public university hospital. The patients were randomly divided into development (*n* = 3108) and validation (*n* = 1602) samples for the building and testing of the nomogram, respectively. Multivariate logistic regression was developed on the basis of pre-pregnancy clinical covariates assessed for their association with CI occurrence.

**Results:**

A total of 109 patients (2.31%) experienced CI among all the enrolled patients. Body mass index (BMI), basal serum testosterone (T), gravidity and uterine length were associated with CI occurrence. The statistical nomogram was built based on BMI, serum T, gravidity and uterine length, with an area under the curve (AUC) of 0.84 (95% confidence interval: 0.76–0.90) for the developing cohort. The AUC for the validation cohort was 0.71 (95% confidence interval: 0.69–0.83), showing a satisfactory goodness-of-fit and discrimination ability in this nomogram.

**Conclusion:**

The user-friendly nomogram which graphically represents the risk factors and a pre-pregnancy predicted tool for the incidence of CI in patients undergoing IVF/ICSI treatment, provides a useful guide for medical staff on individualized decisions making, where preventive measures could be carried out during the IVF/ICSI procedure and subsequent pregnancy.

**Supplementary Information:**

The online version contains supplementary material available at 10.1186/s12958-020-00693-x.

## Background

Cervical insufficiency (CI) is a clinical diagnosis used to describe second trimester painless pregnancy loss in the absence of a precipitating cause. The incidence rate of CI is around 1–2% in all pregnancies, however, up to 15% of pregnancy losses during 16–28 weeks could be ascribed to CI [1, 2]. As one of the important factors in preterm birth (PTB), an increased incidence of CI has become a serious obstacle to healthy delivery and complication-free postpartum period for both mother and their fetus [3].

The cervix remains closed during pregnancy and undergoes a simultaneously progressive physiological remodeling with serially changing competence to prepare for labor. Cervical ripening is a crucial process in cervical remodeling [4]. In cases of CI, the cervix dilates without painful uterine contraction, leading to inability of the cervix to remain closed until a term pregnancy. Although cervical weakness may be associated with a variety of events including cervical ablation, cervical excision or cervical hypoplasia after diethylstilbestrol, [5] most women diagnosed with CI actually have normal cervical integrity [6]. On the other hand, premature cervical ripening which can also be caused by subclinical infection, local inflammation, hormonal effects or genetic factors, is now the generally accepted cause of CI [4, 5]. The initial signs in patients with CI are usually present before the experience of contractions or any other clinical symptom of miscarriage or preterm labor, so the opportunity of providing interventional treatment is limited. Emergency cervical cerclage is sometimes performed in patients with cervical dilation, but infections and postoperative contractions with subsequent miscarriage may also happened. There is still a lack of a well-defined population for whom the procedure is clearly beneficial [7].

In 2011, routine cervical ripening recording was recommended by the Global Alliance to Prevent Prematurity and Stillbirth, [8] where a cervix shorter than 25 mm was regarded as the best predictor of PTB due to the inverse relationship between mid-trimester cervical length and gestational age at delivery [9, 10]. However, in the preterm prediction study, among all the women with a short cervix, only 27% of them delivered before 37 weeks, and less than 18% women delivered before 35 weeks [10]. Extremely short cervix less than 15 mm only conferred a 50% chance of delivering prior to 33 weeks, [11] so cervical length showed limited predictive power as single indicator. There were also studies which revealed a crucial role of androgens in regulating cervical function and PTB, but failed to provide clinical evidence of consistent relationship between androgen excess and CI [12, 13].

In vitro fertilization (IVF) and intracytoplasmic sperm injection (ICSI) have been extensively applied as important assisted reproductive technologies in the past 30 years. However, IVF/ICSI treatment has been reported to be accompanied with increased pregnancy-related complications besides adverse perinatal outcomes [14, 15]. Minimal amounts of published data are available regarding the rate of CI or its risk factors in women conceived by IVF/ICSI procedure, although CI is a significant cause of miscarriage/ preterm birth. In this aspect, exploration of the potential risk factors contributing to CI prior to conception and further setting up of a predictive calculation model with a combination of all the risk factors will be of great importance. The results will be beneficial for medical staff to take preventive measures to minimize fetal loss after IVF/ICSI treatment besides aiding the decision-making process.

Therefore, the aims of this study were to investigate the risk factors of CI in patients who had their first intrauterine pregnancy after IVF/ICSI treatment, and to set up a nomogram model based on retrospective data analysis to predict the incidence of CI.

## Patients and methods

### Data sources

All women who conceived during 2011 and 2018 by IVF/ICSI treatment at The Sixth Affiliated Hospital of Sun Yat-sen University, Guangzhou, China, were screened for this retrospective cohort study. Women with singleton or twin pregnancy were all eligible for inclusion. Exclusion criteria included congenital uterine malformation (unicornuate, bicornuate, septate uterus) and potential cervical damage due to previous peritoneal chemotherapy, radiation or cervical surgery (cervical conization, cervical tear or laceration, trachelectomy, etc.). Indications for IVF/ICSI included the tubal factor, male factor, incretion and immunity factor. Demographic data on age, body mass index (BMI), infertility duration, gravidity and parity, IVF/ICSI cycle, basal sexual hormone levels, uterine length (defined as the distance from the internal cervical os to the uterine fundus, measured at patients’ first visit before IVF/ICSI treatment) were obtained from the clinical database.

The diagnosis of CI was ascertained by detailed chart review, including visit notes, ultrasound and operative reports, as well as pathology and microbiology reports to exclude patients with cervical changes attributable to infection. The diagnosis was defined by the presence of painless cervical dilation (according to traditional ACOG criteria) [16] or progressive cervical shortening with funneling to a residual cervix of ≤ 2.5 cm before 36 weeks of gestation [5]. Patients were firstly diagnosed with CI in the pregnancy. Gestational age was based on the day of embryonic transfer. All identified CI cases were confirmed by experienced perinatologists. Cases with insufficient data, selective fetal reduction, infection, preterm labor and preterm premature rupture of membranes, were not classified as CI in this study.

### Data analysis

Different descriptions were used in Table 1 based on the types of statistics. Specifically, statistics such as age, BMI, FSH etc., which had a Gaussian distribution were presented as mean ± SD. On the other hand, data such as twin rate, CI rate etc., which were categorical variables were described as absolute frequencies. We used the Youden Index to calculate the optimal cut-off point of uterine length related to the occurrence of CI. To develop and validate the prognostic nomogram, enrolled patients were divided into a development set (*n* = 3108) and validation set (*n* = 1602) by the sampling techniques of random numbers. Patients with missing values on any of the analyzed predictors were excluded.

**Table 1:** Patient characteristics in the training and the validation cohorts

Characteristics^a^	Developing set *n* = 3108	Validating set *n* = 1602	*P*
CI occurrence, *n* (%)	69 (2.22)	40 (2.50)	0.549
Age, years	31.44 ± 4.21	31.66 ± 4.26	0.722
Infertility duration, years	4.41 ± 2.96	4.43 ± 3.00	0.981
Twins pregnancy, *n* (%)	410 (13.19)	208 (12.98)	0.841
BMI, kg/m^2^	22.00 ± 3.05	22.04±3.07	0.641
FSH, IU/L	6.73 ± 1.83	6.72 ± 1.99	0.385
LH, IU/L	5.27 ± 2.68	5.34 ± 2.87	0.822
E2, pg/mL	39.17 ± 13.67	39.83 ± 13.64	0.080
T, ng/mL	0.33 ± 0.24	0.33 ± 0.24	0.923
Uterine length > 45 mm, *n* (%)	785 (25.26)	419 (26.15)	0.504
IVF/ICSI cycle			
1	1346 (43.31)	667 (41.63)	
2 or 3	976 (31.40)	540 (33.71)	
> 3	786 (25.28)	395 (24.65)	0.272
Median, (range)	2 (1–4)	2 (1–4)	0.153
Gravidity, *n* (%)			
< 1	1656 (53.28)	833 (52.00)	
1 or 2	1239 (39.86)	655 (40.89)	
> 2	213 (6.85)	114 (7.12)	0.701
Median, (range)	0 (0–4)	0 (0–4)	0.306
Hysteroscopic surgery, *n* (%)			0.064
Yes	1231 (39.61)	672 (41.94)	
No	1877 (60.39)	930 (58.06)	

*Abbreviations: BMI,* body mass index*; FSH,* follicular stimulating hormone*; E2,* estrogen*; T,* testosterone; *IVF/ICSI,* in vitro fertilization/ intracytoplasmic sperm injection*; CI,* cervical insufficiency

^a^ Continuous variable are expressed as mean ± standard deviation, SD, categorical variables as absolute frequencies, *n* (%)

### Development of the model

We developed a nomogram to predict patient-specific likelihood of CI occurrence in women who have undergone IVF/ICSI treatment by using the developing cohort of 3018 patients. The end-point of the study was the incidence rate of CI after IVF/ICSI treatment. Correlation analysis had been performed to identify linear relationship between every predictor variable and CI in developing cohort. Multivariable logistic regression (MLR) analysis was performed using the logistic regression model which included the variables that were significant at univariable analysis (*P* < 0.05) (Table S1). MLR was used to generate coefficients for each variable and the constant in the eq. A nomogram was constructed to be a graphical representation of the prediction model with R software.

Backward variable selection was performed to determine independent predictors. Variables entered into the model were: twin pregnancy, basal serum testosterone (T) levels, patient’s BMI, uterine length and times of gravidity. Variables were eliminated from the model if their removal objectively improved the overall quality of the model (as measured by the Akaike information criterion). The *P* values in the multivariable analysis were based on the Wald test. A *P* value of less than 0.05 was considered significant. Values for each of the model covariates were mapped to points on a scale ranging from 0 to 100. The total points obtained for each model corresponded to the probability of CI.

### Evaluation of the model

The performance of the nomogram was quantified with respect to calibration and discrimination by using the cohort of 1602 patients (validation set) for external validation. A bootstrapping technique to obtain relatively unbiased estimates (2000 repetitions) was performed. For each group of 2000 bootstrap samples, the model was refitted and tested against the observed sample in order to estimate the predictive accuracy and bias. The predictive accuracy of the models provides an estimate of the average optimism of the area under the curve (AUC), quantifying the level of agreement between the predicted probabilities and the actual possibility of having the event of interest. Calibration was studied from graphical representations of the relationship between the observed outcome frequencies and the predicted probabilities.

Statistical analyses were performed using the STATA data analysis and statistical software version 14 MP and Regression Modeling Strategies (RMS, R version 3.6.3). For the nomogram establishment and the AUC measurements, we used the “regplot”, “pROC” and “rms” in R software. Differences between groups were compared using Student’s *t*-test or Chi-squared test as appropriate.

## Results

### Description of the study population

After applying the inclusion and exclusion criteria of the current study, a total of 4710 patients who conceived by IVF/ICSI procedures from January 2011 to December 2018 were identified as eligible and were analyzed in this study. One hundred-nine patients (2.31%) were diagnosed with CI in the entire cohort. Then, patients were divided into a development set and validation set by the sampling techniques of random numbers. The model was built from a training cohort of 3108 patients and was tested on an independent validation cohort of 1602 patients. Epidemiological, clinical, biological demographics and therapeutic strategies of the development and validation cohorts are summarized in Table 1. No significant difference was observed in the patients’ characteristics between the two cohorts. CI occurred in 69 patients (2.22%) in the development cohort while 40 patients (2.50%) were diagnosed with CI in the validation cohort. Nineteen patients with CI (17.43%) experienced hysteroscopic surgery before pregnancy.

### Logistic regression analysis revealed overweight, increased gravidity, high serum T and smaller uterine length were risk factors of CI occurrence

**Fig. 1:**
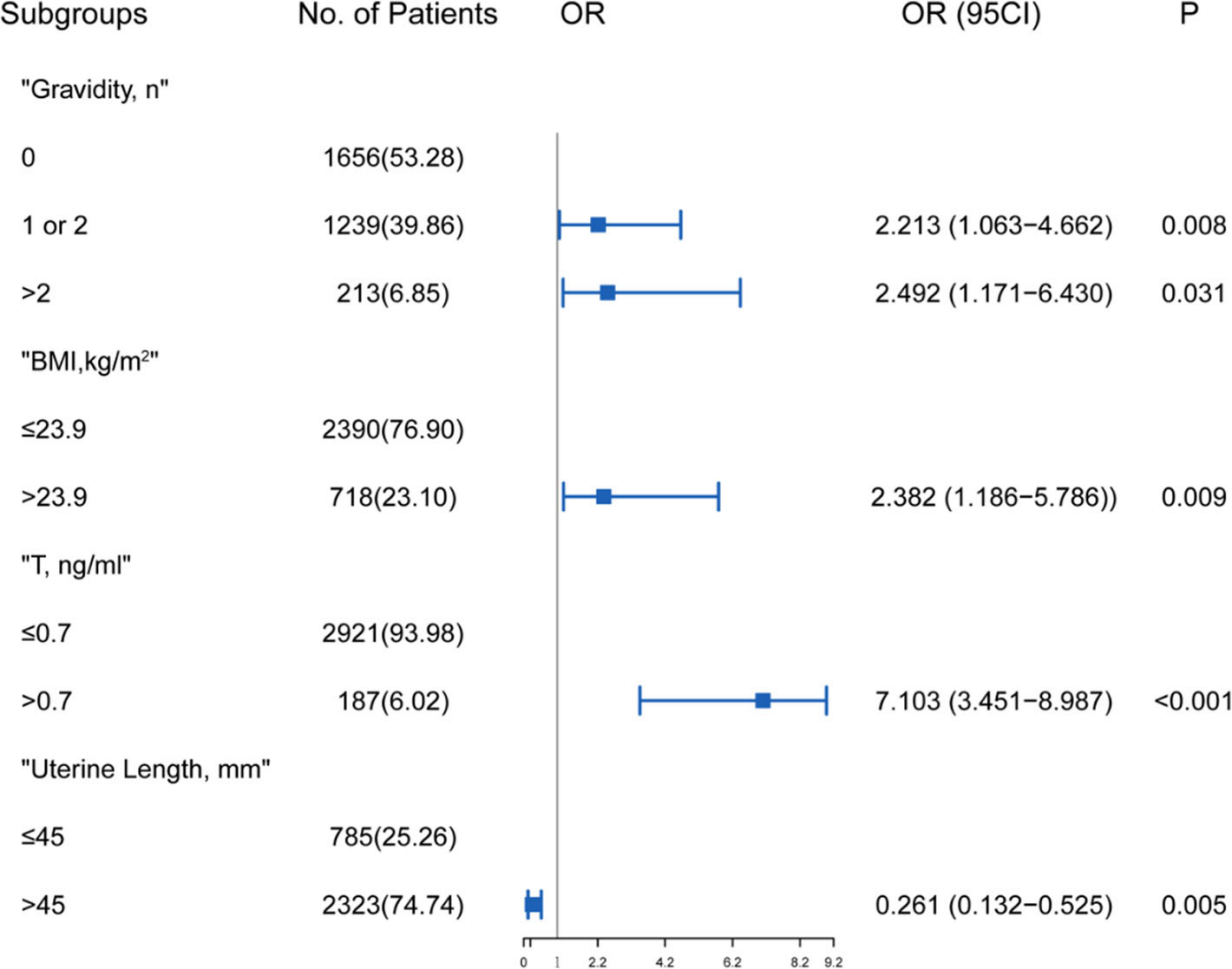
The Forest plot of predictive factors of cervical insufficiency occurrence in Multivariable analysis. Abbreviations: BMI, body mass index; T, testosterone; OR, odds ratio; CI, confidence interval; *P < 0.05 was considered statistically significant

The optimal cut-off point of the uterine length related to the occurrence of CI was 45 mm according to the Youden Index. Table S1 summarizes univariable and multivariable analysis. According to univariable logistic regression analysis, CI occurrence was significantly correlated with gravidity (*P* = 0.023), BMI (*P* = 0.012), uterine length (*P* < 0.001) and serum T (*P* < 0.001). In the multivariable analysis of the developing cohort, probability of CI was significantly correlated with serum T (odds ratio [OR], 7.10; 95% CI, 3.45–8.99; *P* < 0.001), BMI (OR, 2.38; 95% CI, 1.19–5.79; *P* = 0.009), uterine length (OR, 0.26; 95% CI, 0.13–0.53; *P* = 0.005) and gravidity (*P* = 0.031) (Fig. 1). T > 0.7 ng/mL, uterine length < 45 mm, increased BMI and gravidity were associated with an increased occurrence rate of CI.

### Development of the models from the derivation cohort

**Fig. 2:**
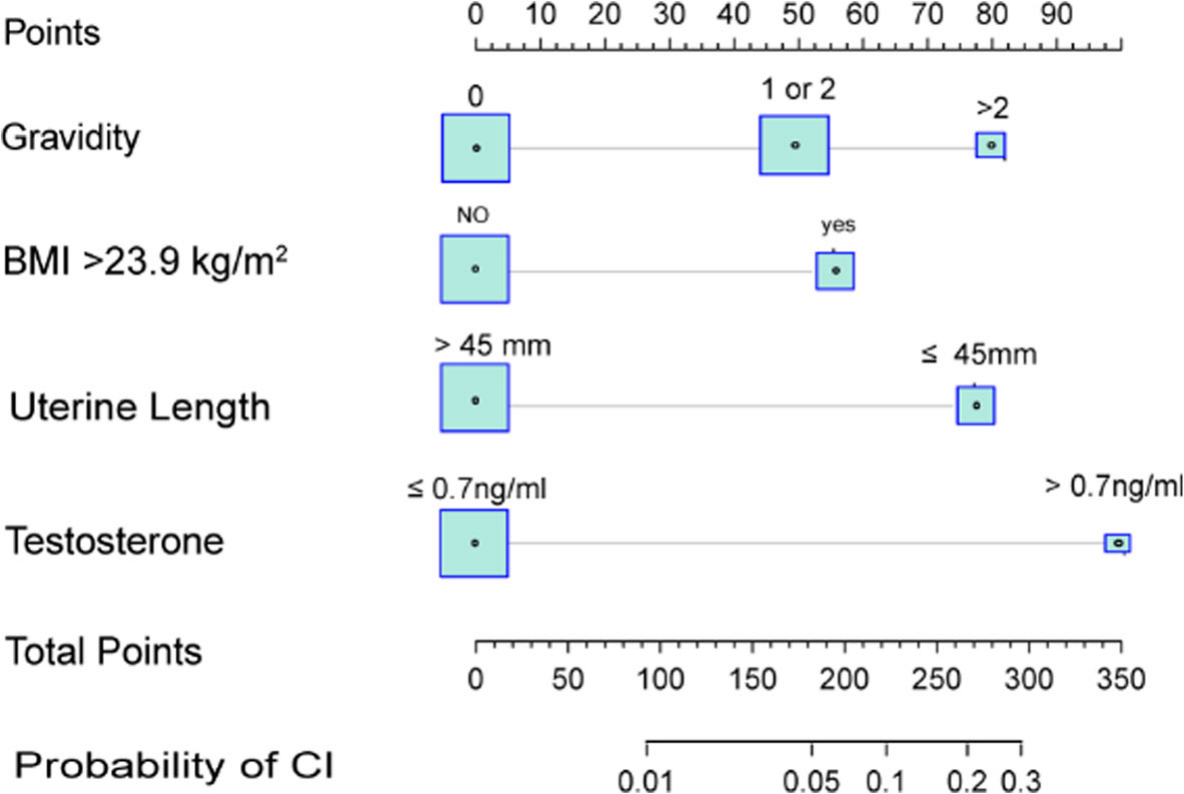
Nomogram to predict cervical insufficiency rate in patients undergoing IVF/ICSI. The total score calculated using serum T, gravidity, BMI and uterine length corresponds to the probability of CI. Abbreviations: BMI, body mass index; IVF/ICSI, in vitro fertilization/ intracytoplasmic sperm injection; CI, cervical insufficiency

On the basis of the univariable and multivariable logistic regression analysis we performed, a nomogram incorporating the significant risk factors was established to predict the probability of CI occurrence (Fig. 2). A total score was calculated using serum T, gravidity, BMI and uterine length. The equation describing the probability of CI occurrence was: *P* = 1/(1 + exp. (−X)), where X= − 5.459811 + 0.9332314 × *V1*+ 2.128771 × *V2*–1.144699 × *V3* + 0.6665644× *V4*, where *V1* was BMI (1 if > 23.9 kg/m^2^ and 0 if ≤ 23.9 kg/m^2^), *V2* serum T level (1 if > 0.7 ng/mL and 0 if ≤ 0.7 ng/mL), *V3* the uterine length (1 if > 45 mm and 0 if ≤ 45 mm) and *V4* the gravidity (2 if > 2, 1 if 1 or 2 and 0 if < 1). The nomogram derived from this equation is reported in Fig. 2.

### Validation of predictive accuracy

**Fig. 3:**
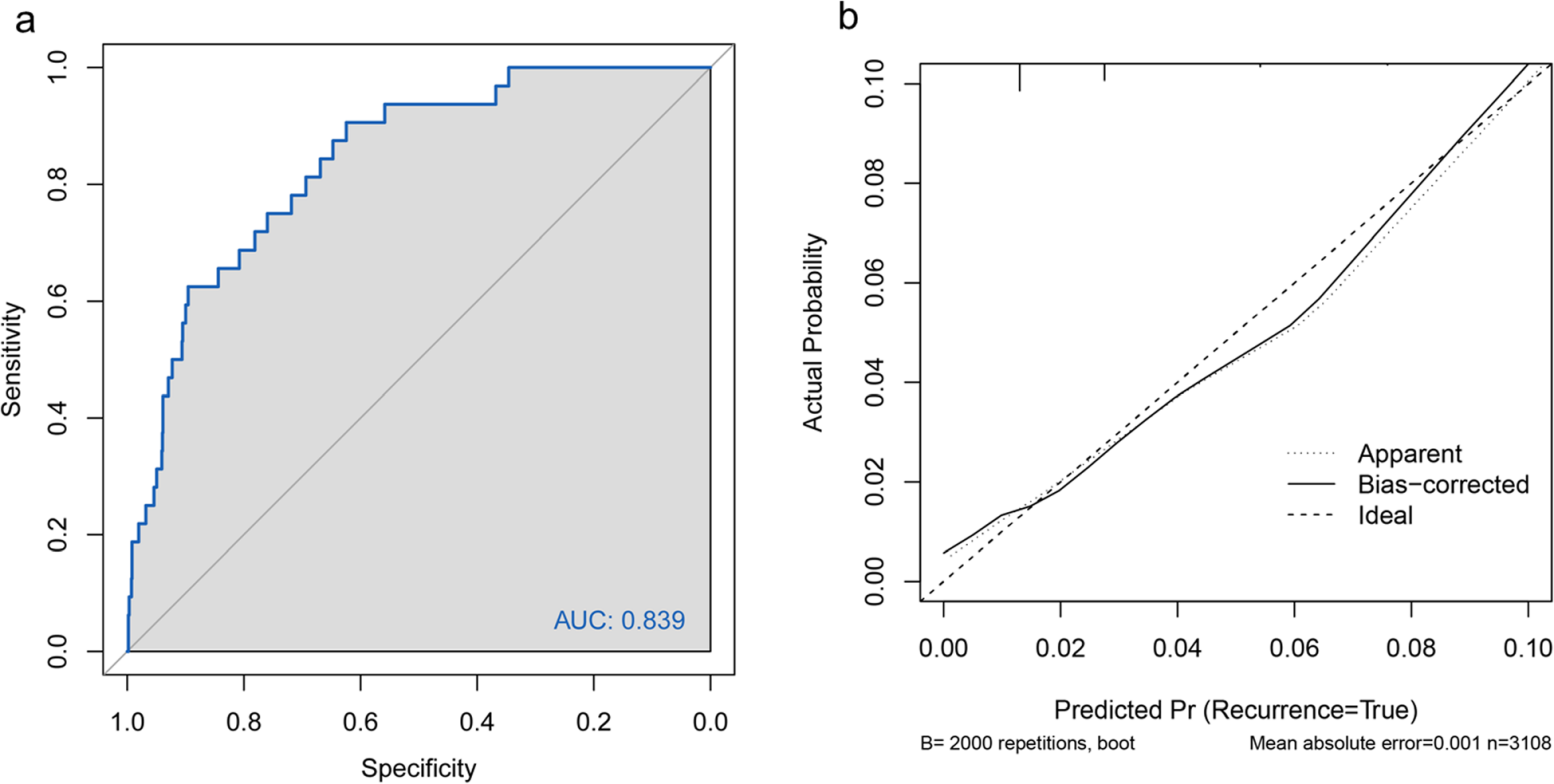
Discrimination and calibration of a model to predict cervical insufficiency rate in patients undergoing IVF/ICSI. **a**: ROC curve of a model to predict cervical insufficiency rate in patients undergoing IVF/ICSI. **b**: Calibration curve showing the association between the probability of CI as predicted by the model and the observed CI rate in the developing set. Abbreviations: IVF/ICSI, in vitro fertilization/ intracytoplasmic sperm injection; CI, cervical insufficiency; ROC, Receiver Operating Characteristic Curve

No significant difference was observed between the predicted probability obtained from the bootstrap correction and the actual probabilities of CI occurrence (*P* = 0.261), which implied that the nomogram was well calibrated. The model demonstrated an AUC of 0.84 (95% confidence interval: 0.76–0.90) in the training cohort (Fig. 3 a & b), which denoted good performance. The AUC of the receiver operating characteristic (ROC) curve in the validation set was 0.71 (95% confidence interval: 0.69–0.83) which indicated fair performance.

## Discussion

To the best of our knowledge, the nomogram we present here is the first to predict individual CI occurrence rate. Data analysis of 4710 infertile patients who have undergone IVF/ICSI procedure resulted in an original nomogram which could predict the likelihood of the occurrence of CI based on data with remarkable clinical significance. The nomogram was developed in a training cohort which included 3108 patients and was tested on an external independent validation cohort which included 1602 patients. Performance was evaluated using both calibration and discrimination. The value of the model lies in the combination of covariate data that was easily accessible which originated from clinical, biological and imaging characteristics, including: pre-pregnancy BMI, serum T, uterine length and gravidity. The results of the derived nomogram provide a graphically simple and straightforward calculator which is of particular interest for clinicians to make an informed decision on the number of embryos to transfer or preventive measures to be taken in early pregnancy.

In our study, we found that basal serum T was a significant risk factor to predict CI incidence. In the research of Dawood et al., a notable increase in serum T was observed at the third trimester of normal pregnancy [17]. Previous studies have found that androgens were vital for cervical remodeling and for the promotion of cervical ripening by altering the collagenase activity and thus decreasing fibrillar collagen organization [13]. Thus, a circulating increase of androgens throughout the course of pregnancy probably resulted in improper remodeling and advanced timing of cervical ripening, which gave rise to CI with adverse consequences of preterm birth and miscarriage. The most common hyperandrogenic conditions are known as ‘non-tumour ovarian hyperandrogenism’ which includes Polycystic Ovarian Syndrome (PCOS) and hyperreactioluteinalis (HL) [18]. HL, which predominantly occurs during the second or third trimester, is highly associated with adverse pregnancy outcomes such as preterm birth [19, 20]. Patients with PCOS with hyperandrogenism were reported to have a higher prevalence of CI and an approximately 6% higher risk of preterm delivery compared to women without PCOS [21, 22]. Though some studies emphasized that increased androgen levels might be crucial for the maintenance of pregnancy, the mechanism by which androgens impacted pregnancy outcomes has not been fully understood. Further investigation of the underlying mechanisms of androgen action on cervical remodeling is still needed.

Another risk factor of the occurrence of CI is uterine length which was less than 45 mm as revealed by MRL. Routine checks for uterine size before Artificial Reproductive Therapy (ART) is beneficial for detecting patients at an increased risk. In this retrospective study, the length of corpus uteri was recorded as the distance from the internal cervical os to the uterine fundus. Previous studies have supported that abnormal development of the corpus uteri impacted the development of cervix uteri [23]. A prospective study by Hawkins et al. revealed that women with uterine lengths (defined as the distance from the external cervical os to the uterine fundus) shorter than 6 cm were more likely to experience spontaneous abortions [24]. Collectively, the use of uterine length as a significant determinant factor in pre-pregnancy examinations should never be ignored. In addition, the increased weight may increase pressure on the cervix. Frezza et al. measured opening abdominal pressures in patients with varying BMI values by connecting a Verress needle to a pressure monitor, where the results proved that every increase of 1 kg/m^2^ in BMI was accompanied with a 0.07 mmHg increase of average abdominal pressure [25]. Thus, the increased abdominal pressure may be transmitted to the cervix and facilitated the occurrence of CI. Consistently, BMI was a strong correlative factor in our model. Proper weight management would be beneficial to women with BMIs higher than 23.9 kg/m^2^ before IVF/ICSI treatment.

Our data revealed the CI incidence rate of 2.31% in the chosen population, which was higher than the reported CI rate in the general obstetric population [1]. Some studies also reinforced that an increased relative risk of CI had been associated with IVF/ICSI procedure [26, 27]. This was probably because these patients usually experienced more intrauterine surgical intervention during IVF/ICSI procedure, which were potential risk factors for cervical injury. Besides, our study showed that increased gravidity was a significant risk of CI occurrence. It is known that labor, especially which experienced precipitous deliveries and difficult deliveries, impacts cervical competence in varying degrees. On the other hand, our study was limited by the relatively small number of patients with twin pregnancies, where the correlation with CI was insignificant. Nonetheless, single embryo transfer is encouraged for fear of the adverse outcomes of multiple pregnancy. Combined with previous studies and our results, women with PCOS who are known to be associated with hyperandrogenism, overweight, insulin resistance and subfertility, may be at a higher risk of CI and should garner the focused attention of clinicians.

Still, some limitations of the present study have to be underlined. First, a lack of consensus criteria and advances in molecular and imaging technology have contributed to the challenges in the consistency of the diagnosis of CI in this study. Second, as a retrospective study, we lose the records of the cervical length before pregnancy and the circulating T concentration along with the other types of androgens in mid-trimester pregnancy which could provide more clinical details in the association between androgens and CI. Third, the retrospective nature of the study cannot exclude all biases.

Despite these limitations, our results provide the clinical evidence of the correlation between androgen excess and CI occurrence. Our nomogram model to predict the probability of CI occurrence could be a useful tool in aiding physicians and patients undergoing the IVF/ICSI procedure to decide on embryo-transfer option and to pay special attentions during prenatal visits. Additionally, we highlighted that the treatment of hyperandrogenism before pregnancy would be beneficial for a healthy newborn.

## Conclusion

In conclusion, our analysis presents a user-friendly model to predict the incidence rate of CI prior to IVF/ICSI treatment. Relative risk assessment could be performed during infertility consultation and appropriate measures could be carried out in advance to minimize the probability of fetal loss. Furthermore, our results support the concept that androgen excess negatively impacts cervical competence and draws attention to certain populations of infertile women (especially women with PCOS) with risk factors.

## Supplementary Information

**Additional file 1: Table S1.** Univariate and multivariate analysis of factors predicting the CI occurrence in patients undergoing IVF/ICSI.

## Data Availability

Not applicable.

## Abbreviations

CI: Cervical insufficiency
IVF/ ICSI: In vitro fertilization/ Intracytoplasmic sperm injection
BMI: Body mass index
T: Testosterone
AUC: Area under the curve
PTB: preterm birth
FSH: Follicle stimulating hormone
MLR: Multivariable logistic regression
ROC: Receiver operating characteristic
PCOS: Polycystic Ovarian Syndrome
HL: Hyperreactioluteinalis
ART: Artificial reproductive therapy
