# Supplementary material for: Development and validation of a model for individualized prediction of cervical insufficiency risks in patients undergoing IVF/ICSI treatment
Source: Reprod Biol Endocrinol. 2021 Jan 7;19:6. doi: 10.1186/s12958-020-00693-x (PMC7789534; doi:10.1186/s12958-020-00693-x)
Supplement: Supplementary file 1 — Additional file 1: Table S1. Univariate and multivariate analysis of factors predicting the CI occurrence in patients undergoing IVF/ICSI. [file 12958_2020_693_MOESM1_ESM.docx]

Table S1. Univariate and multivariate analysis of factors predicting the CI occurrence in patients undergoing IVF/ICSI

|  | Univariate analysis | | Multivariate analysis | |
| --- | --- | --- | --- | --- |
|  | OR  95% CI | *P* value | OR  95% CI | *P* value |
| Age | 1.036 (0.984-1.089) | 0.178 |  |  |
| IVF/ICSI cycle | 2.311 (0.95-3.41) | 0.673 |  |  |
| Infertility duration | 0.996 (0.925-1.073) | 0.925 |  |  |
| Uterine length >45 mm | 0.455 (0.292-0.710) | <0.001* | 0.261 (0.132-0.525) | 0.005* |
| FSH | 0.847 (0.672-1.068) | 0.757 |  |  |
| E2 | 1.016 (0.99-1.041) | 0.597 |  |  |
| LH | 1.088 (0.983-1.205) | 0.105 |  |  |
| T > 0.7 ng/mL | 6.098 (3.227-11.526) | <0.001* | 7.103 (3.451-8.987) | <0.001* |
| BMI  >23.9 kg/m^2^ | 2.315 (1.480-3.621) | 0.012* | 2.382 (1.186-5.786) | 0.009* |
| Twin pregnancy | 1.221 (0.468-3.189) | 0.683 | 0.731 (0.283-1.956) | 0.537 |
| Gravidity |  | 0.023* |  | 0.031* |
| <1 | Reference |  | Reference |  |
| 1 or 2 | 1.908 (1.148-3.172) |  | 2.213 (1.063-4.662) |  |
| >2 | 2.762 (1.045-4.214) |  | 2.492 (1.171-6.430) |  |
| Hysteroscopic surgery |  | 0.157 |  |  |
| Yes | Reference |  |  |  |
| No | 0.746 (0.251-3.183) |  |  |  |

*Abbreviations: BMI, body mass index; FSH, follicular stimulating hormone; LH, luteinizing hormone E2, estrogen; T, testosterone; IVF/ICSI, in vitro fertilization/ intracytoplasmic sperm injection; *P < 0.05 was considered statistically significant*
